# Purification, Characterization, and Mode of Action of Pentocin JL-1, a Novel Bacteriocin Isolated from* Lactobacillus pentosus*, against Drug-Resistant* Staphylococcus aureus*

**DOI:** 10.1155/2017/7657190

**Published:** 2017-11-29

**Authors:** Han Jiang, Jiong Zou, Hui Cheng, Jiehong Fang, Guangrong Huang

**Affiliations:** Key Laboratory of Marine Food Quality and Hazard Controlling Technology of Zhejiang Province, College of Life Sciences, China Jiliang University, Hangzhou, Zhejiang 310018, China

## Abstract

*Staphylococcus aureus* and its drug-resistant strains, which threaten public health and food safety, are in need of effective control by biopreservatives. A novel bacteriocin, pentocin JL-1, produced by* Lactobacillus pentosus* that was isolated from the intestinal tract of* Chiloscyllium punctatum*, was purified by a four-step chromatographic process. Mass spectrometry based on MALDI-TOF indicated that pentocin JL-1 has a molecular mass of 2987.23 Da. Only six of the twenty-five amino acids could be identified by Edman degradation. This bacteriocin is thermostable and tolerates a pH range of 5–7. Also, it is sensitive to proteinase K, trypsin, pepsin, and alkaline protease. This bacteriocin has a broad inhibitory spectrum against both Gram-positive and Gram-negative strains and in particular is effective against multidrug-resistant* S. aureus*. Additionally, we showed that the cell membrane is the target of pentocin JL-1 against methicillin-resistant* S. aureus* (MRSA), causing a loss of proton motive force. Furthermore, pentocin JL-1 has a drastic impact on the structure and integrity of MRSA cells. These results suggest that pentocin JL-1 has potential as a biopreservative in the food industry.

## 1. Introduction

*Staphylococcus aureus *belongs to the Gram-positive Micrococcaceae family and is one of the most serious bacterial pathogens globally [1]. It can produce several toxins including staphylococcal enterotoxins, which are a major cause of several illnesses, especially food-borne diseases resulting from the consumption of a broad variety of contaminated food such as meats, dairy products, baked goods, and salads [1–3]. In 1961, methicillin-resistant* S. aureus *(MRSA) was first found among* S. aureus *clinical isolates [4] and carries an increased risk for morbidity and mortality. Moreover, the preferred treatment agent against MRSA, vancomycin, has been reported to have reduced efficacy [5]. In addition, many other types of multidrug-resistant* S. aureus *have been detected in the past few years [6–8]. Drug-resistant* S. aureus *strains are a potential risk to humans as they could transfer the resistance to other pathogenic bacteria of humans through the food chain, the genetic pool of bacteria, bacteriophages, or DNA fragments [9, 10]. There is thus an urgent need to discover novel and effective biopreservatives and antimicrobial drugs to inhibit* S. aureus *and its drug-resistant strains for either food preservation or prevention and control of bacterial infectious diseases [10].

Bacteriocins are prokaryotic proteins or peptides, which exhibit inhibitory activity against other prokaryotes [10, 11]. Particularly, the bacteriocins produced by lactic acid bacteria (LAB) have been the focus of much research because LAB and their metabolic products are generally regarded as safe (GRAS) [12] and have potential application as natural preservatives in the food industry [13]. Currently, bacteriocins produced by Gram-positive strain are classified into five groups [14]: class I, small (<5 kDa) and linear peptides containing posttranslationally modified amino acids, including those with thioether bridges formed between the thiol groups of Cys residues and the *β*-carbon of other amino acid residues; class II, small (<10 kDa), linear peptides without posttranslationally modified amino acids; class III, proteins (>10 kDa); class IV, small (<10 kDa), circular peptides without posttranslationally modified amino acids and with an amide bond between the N- and C-termini; class V, small (<5 kDa), linear or circular peptides containing extensively posttranslationally modified amino acids with thioether bridges formed between *α*-carbon of other amino acid residues and the thiol groups of Cys residues. However, there is no international standard of classification, and other schemes have been proposed along with information regarding their characteristics [15, 16].

In recent years, many useful LAB bacteriocins have been identified and studied, such as lactococcin A [17], pentocin TV35b [18], amyovorin L471 [19], lacticin Q [20], plantaricin ZJ008 [11], and lactocin XN8-A [21]. So far, nisin produced by* Lactococcus lactis*, pediocin produced by* Pediococcus acidilactici*, and a combination of three bacteriocins (carnocyclin A, carnobacteriocin BM1, and piscicolin 126), all produced by* Carnobacterium maltaromaticum* UAL307, which has been commercialized in the USA and Canada, with the name of Micocin®, are used as food preservatives commercially [22, 23]. Other effective LAB bacteriocins are in the process of obtaining commercial status to be used as food preservatives [22].

Treatment with LAB bacteriocins is an effective and safe way to inhibit* S. aureus* growth in food. Many researchers have shown that LAB bacteriocins have anti-MRSA ability [11, 24, 25]. However, only a few studies have been performed to investigate the LAB bacteriocins against other drug-resistant* S. aureus *such as anticiprofloxacin, anticefoxitin, and antigentamicin [21]. In our study, we aimed to purify and characterize pentocin JL-1, which was produced by* Lactobacillus pentosus *isolated from the intestinal tract of* Chiloscyllium punctatum*, exhibiting a broad inhibitory spectrum. This bacteriocin can inhibit not only MRSA but also other multidrug-resistant* S. aureus *strains. In addition, the mode of action by which pentocin JL-1 causes cell membrane damage in MRSA was characterized.

## 2. Materials and Methods

### 2.1. Isolation and Identification of Antimicrobial Strains

The intestinal tracts of* C. punctatum* (grey carpet shark) were dissected and homogenized in 20 mL saline solution under sterile conditions and were then plated in serial dilutions in deMan, Rogosa, and Sharpe (MRS) medium. The plates were incubated aerobically at 30°C for 24 h. Several colonies were picked at random and incubated again in MRS broth. For screening for bacteriocin-producing strains, the agar-well diffusion test was used to detect antimicrobial activity in cell-free supernatants (filtered through a 0.22 *μ*m Millipore filter) obtained by centrifugation (10,000*g*, 30 min, 4°C) after 18, 36, 60, and 72 h incubation [10]. The Gram-positive strain MRSA GIM 1.771 and the Gram-negative strain* Escherichia coli* O157:H7 GIM 1.707 were used as indicator strains. Before the experiments, the indicator strains were grown to 10^6^ CFU/mL. Then, 1 mL of the culture was mixed with 100 mL soft agar medium and poured onto individual Petri dishes. Subsequently, 8 mm diameter wells were punched onto the plates, and each well was filled with 100 *μ*L of the cell-free supernatants under sterile conditions. The plates were incubated overnight at their respective optimum temperatures, and the clear zones of inhibition were measured in diameter and indicated the presence of antimicrobial activity. In our study, all bacteria culture media and chemical reagents were supplied by Sigma-Aldrich (USA).

The strain with the highest antibacterial activity against MRSA GIM 1.771 and* E. coli* O157:H7 GIM 1.707 was selected and named JL-1. It was stored at −80°C in MRS broth with 25% (*v/v*) glycerol. The strain JL-1 was then identified by 16S rRNA gene sequencing with the forward primer 5′-AGAGTTTGATCCTGGCTCAG-3′ and the reverse primer 5′-CTACGGCTACCTTGTTACGA-3′. Subsequently, sequence homologies were analyzed by comparing the sequence with those in the NCBI database and phylogenetic analysis was carried out using MEGA 5.0.

### 2.2. Purification of the Bacteriocin

The strain JL-1 was grown in MRS medium at 30°C for 72 h. The ferments were centrifuged (10,000*g*, 30 min, 4°C), and the cell-free supernatant was absorbed with the macroporous resin D4020 (average pore diameter 100–105 Å, specific surface area of 540–580 m^2^/g, Nankai University Chemical Factory, China). The column was eluted with 20% (*v/w*) ethanol and active fractions were collected. The antimicrobial activity of each fraction was determined by measuring the diameter of the inhibition zone around the wells compared with nisin and expressed as international units (IU) per mL [11]. The active fractions were lyophilized using a freeze-dryer (Labconco, USA). The lyophilized powder was dissolved in 20 mM phosphate buffer (pH 7.0) for the next purification step. Then, cation-exchange chromatography and gel filtration were performed using the AKTA Pure 25 (GE, Uppsala, Sweden) chromatography system equipped with a full wavelength UV detector and an automatic collector. The sample was purified using an SP-Sepharose Fast Flow column (XK 16/40, GE) and elution with 0-1 M NaCl in citric acid-phosphate buffer (pH 7.0) at 1.0 mL/min. The active fraction was collected and purified by an Ultrahydrogel TM 250 gel filtration column (Waters, USA), after elution with pure water at 1.0 mL/min. Then, the active fraction was purified on a C_18_ column (10 × 250 mm, 5 *μ*m) using the LC-6AD semipreparative High Performance Liquid Chromatography (HPLC) system (Shimadzu, Japan) at 2.5 mL/min with a gradient elution of 100% buffer A (95% water, 5% acetonitrile, and 0.1% trifluoroacetic acid) to 100% buffer B (100% acetonitrile and 0.1% trifluoroacetic acid). The active fraction was collected and repurified using an analytical C_18_ column (150 × 4.6 mm, 5 *μ*m) with an elution with 40% acetonitrile. The active fraction was collected and lyophilized for mass spectrum (MS) detection. Purified pentocin JL-1 was lyophilized using a freeze-dryer (Labconco, USA). MRSA GIM 1.77 was used as the indicator strain for the activity test.

### 2.3. Mass Spectrometry and Amino Acid Sequence

The molecular mass of the purified pentocin JL-1 was detected by MALDI-TOF-MS (Shimadzu Axima Assurance, Japan), which was analyzed by GL Biochem (Shanghai, China). The N-terminal amino acid sequence of the purified pentocin JL-1 was detected by PPSQ33A automatic sequencing system (Shimadzu, Japan), which was analyzed by Shanghai Sangon Biotech Company, China.

### 2.4. Bacteriocin Activity Assay

The agar-well diffusion test as described previously was used to detect the antimicrobial activity of the purified pentocin JL-1 (15 *μ*g/mL, pH 5.5) [10]. The indicator strains are listed in Tables 1 and 2. LAB strains were grown in MRS broth at 30°C for 16 h. Other Gram-positive indicator strains were grown in Tryptone Soy Broth (TSB) medium at 37°C and the Gram-negative indicator strains were grown in Luria-Bertani broth medium at 37°C.

Additionally, the minimal inhibitory concentration (MIC) of pentocin JL-1 against MRSA GIM 1.771 was tested. Overnight culture of MRSA GIM 1.771 with the concentration of around 2 × 10^6^ CFU/mL was collected and 50 *μ*L of each was grown in 96 well-microtiter plates (Bio-Rad, USA) with different concentrations of pentocin JL-1, ranging from 50 ng/mL to 15 *μ*g/mL, at 37°C for 24 h. Each concentration was done in triplicate. The MIC represents the bacteriocin concentration at which 100% of growth is inhibited measured by the absorbance at 540 nm [22].

### 2.5. Stability against pH, Temperatures, and Enzymes

To determine pH stability, lyophilized purified pentocin JL-1 was dissolved in 0.05% (*w/v*) acetic acid at 15 *μ*g/mL and was adjusted with 1.0 M HCl or 1.0 M NaOH to different pH values of 2.0, 3.0, 4.0, 5.0, 6.0, 7.0, 8.0, 9.0, and 10.0. Then the above samples were incubated at 37°C for 1 h and were adjusted to pH 5.5 with 1.0 M HCl or 1.0 M NaOH.

To determine thermal sensitivity, lyophilized purified pentocin JL-1 dissolved in 0.05% (*w/v*) acetic acid at 15 *μ*g/mL was evaluated at different temperatures (−20°C, 4°C, 30°C, 60°C, and 100°C) for 1 h and also at the autoclaved condition (121°C at 15 psi for 15 min).

To determine enzymatic sensitivity, lyophilized purified pentocin JL-1 dissolved in 0.05% (*w/v*) acetic acid at 15 *μ*g/mL was treated with the following enzymes at 75 *μ*g/mL under their respective optimum pH and temperatures: proteinase K (pH 7.5, 37°C), trypsin (pH 8.0, 37°C), pepsin (pH 2.0, 37°C), and alkaline protease (pH 8.6, 50°C) (all from Sigma-Aldrich, USA). After incubation for 1 h, the bacteriocin-enzyme mixture was boiled for 5 min to inactivate the enzymes.

The residual antibacterial activities of the above samples were calculated using the agar-well diffusion test with MRSA GIM 1.771 as the indicator strain. The area of inhibition was calculated from the diameter of the inhibition zones, and the decrease ratio was displayed as a percentage. Lyophilized pentocin JL-1 dissolved in 0.05% (*w/v*) acetic acid at 15 *μ*g/mL with pH 5.5 was used as a control in all assays.

### 2.6. Mode of Action

#### 2.6.1. Growth Curve and Time-Killing Kinetics

The bacteriostatic or bactericidal mode of action of pentocin JL-1 was tested as described previously by Zhu et al. with some modifications [11]. MRSA GIM 1.771 was cultivated to the exponential phase in 100 mL of TSB culture medium. The lyophilized purified pentocin JL-1 dissolved in 0.05% (*w/v*) acetic acid was added to the cultures at a final concentration of 1x MIC and the same volume of 0.05% (*w/v*) acetic acid was added to the aforementioned media as a control. Samples were incubated at 37°C and bacterial suspensions were taken each hour for 24 h, and the absorbance was measured at OD_600_. In addition, the viable cell counts on TSB agar medium after the addition of 1x MIC, 2x MIC, and 3x MIC of pentocin JL-1 were also quantified every 10 min for 1 h.

#### 2.6.2. Proton Motive Force (PMF)

To test the effect of pentocin JL-1 on membrane integrity, the cell PMF was assayed. PMF includes the transmembrane electrical potential (ΔΨ) and the transmembrane pH gradient (ΔpH) [26]. ΔΨ was monitored by the fluorescent probe 3,3′-diethylthiadicarbocyanine iodide DisC_2_(5) (Sigma, USA). MRSA GIM 1.771 cells were grown to the exponential phase in 50 mL TSB culture medium, harvested, and washed twice with 50 mL buffer A (250 mM glucose, 5 mM MgSO_4_, 10 mM K_3_PO_4_, and 100 mM KCl, pH 7.0) at 4°C, resuspended in 5 mL of the same buffer and stored on ice for the fluorescence measurements. Then, the cells were added to a fluorescence cuvette together with 0.5 *μ*M DisC_2_(5). The fluorescence emission was monitored at room temperature using an F-4600 spectrofluorometer (Hitachi, Japan) with an excitation wavelength (Ex) of 647 nm and emission wavelength (Em) of 680 nm for 400 s. When the reduction of fluorescence was stable, final concentrations of 1x, 2x, and 3x MIC of pentocin JL-1 were added to the cuvette, respectively, and 0.05% (*w/v*) acetic acid was added as a negative control. Full dissipation of the membrane potential was indicated by addition of 1% Triton X-100. To the control, an equivalent volume of 3x MIC of pentocin JL-1 was added.

ΔpH was monitored by the fluorescent pH probe 2′,7′-bis-(2-carboxyethyl)-5-(and-6)-carboxyfluorescein, acetoxymethyl ester (BCECF AM) (Beyotime, China). MRSA GIM 1.771 cells were grown to the exponential phase in 50 mL TSB culture medium, harvested, washed twice with 50 mL 5 mM HEPES buffer at 4°C, and resuspended in 5 mL of the same buffer for incubation on ice for 1 h. Subsequently, the 1 *μ*M BCECF AM pH probe was added and the solution was incubated at 37°C for 1 h in the dark. Then 1 mL of the incubated solution was added to a fluorescence cuvette together with a final concentration of 1x, 2x, and 3x MIC of pentocin JL-1, respectively. Acetic acid (0.05%,* w/v*) was used as a negative control and 1% (*w/v*) Triton X-100 was used as a positive control. To the control, an equivalent volume of 3x MIC of pentocin JL-1 was added. The fluorescence intensity was monitored at 50 s intervals for 400 s immediately after mixing at Ex 488 nm and Em 535 nm using an F-4600 spectrofluorometer (Hitachi, Japan).

#### 2.6.3. Scanning Electron Microscopy (SEM)

MRSA GIM 1.771 cells in exponential phase were supplemented with 1x MIC of pentocin JL-1 and incubated at 37°C for 10 min. Cells without pentocin JL-1 were used as controls. Cells were collected by centrifugation at 4°C, 6000*g* for 5 min, and washed gently with 500 *μ*L phosphate buffer saline (PBS, 0.1 M, pH 7.4) twice. Subsequently, the cells were fixed in 2.5% glutaraldehyde at 4°C for 16 h and washed gently with 500 *μ*L PBS twice. Then the cells were dehydrated with gradient ethanol solutions (30%, 50%, 70%, 80%, 90%, and 100%) at 4°C and centrifuged at 6000*g* for 15 min. The cells were then freeze-dried using a freeze-dryer (Labconco, USA), coated with gold, and imaged using an SU8010 scanning electron microscope (Hitachi, Japan).

### 2.7. Statistical Analysis

All related experiments were done in triplicate and the results are expressed as mean ± standard deviation. Data analysis was performed with SPSS 19.0 and Origin 8.0. Comparison of data on stability of pentocin JL-1 against pH, temperatures, and enzymes were performed using independent sample *t*-test and *p* < 0.05 was considered statistically significant.

## 3. Results and Discussion

### 3.1. Identification of the Bacteriocin-Producing Strain JL-1

**Figure 1:**
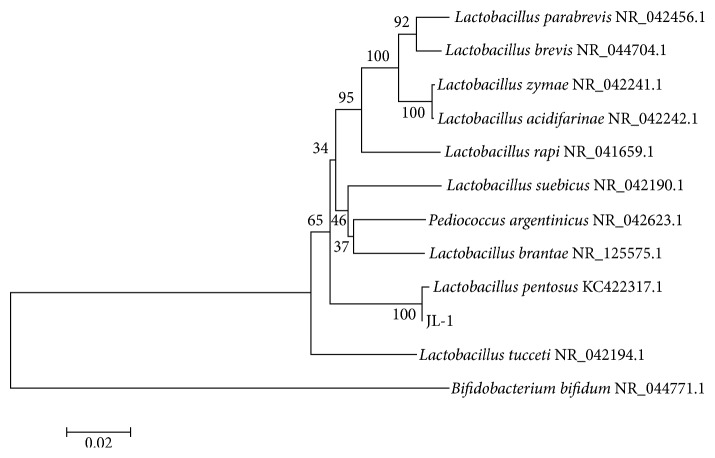
Phylogenetic tree of strain JL-1 based on its 16S rRNA sequence.

The strain JL-1 was selected at 72 h incubation because of its highest antibacterial activity against MRSA GIM 1.771 and* E. coli* O157:H7 GIM 1.707 with a diameter of the inhibition zones of 24.8 ± 0.5 mm and 23.9 ± 0.2 mm, respectively. It is a Gram-positive and catalase-negative* Bacillus*. The 16S rRNA gene sequence was amplified by PCR, and a 1485 bp gene fragment was sequenced after cloning and aligned using the NCBI database. We found that the 16S rRNA sequence of strain JL-1 had 99% similarity with that of* L. pentosus *KC422317.1. Additionally, a phylogenetic tree was constructed using MEGA 5.0 (Figure 1). The sequence of the 16S rRNA gene from strain JL-1 was submitted to the GenBank database under the accession number KY777710.

It has been reported that* L. pentosus*, a* Lactobacillus* species usually isolated from fermented and pickled food and animal intestines, can produce many functional metabolites, such as exopolysaccharides [27], *β*-galactosidase [28], and also some bacteriocins [29–31].* L. pentosus *is LAB, so its metabolic products are GRAS and have the potential to act as natural preservatives [12, 32].

### 3.2. Purification of the Bacteriocin

**Figure 2:**
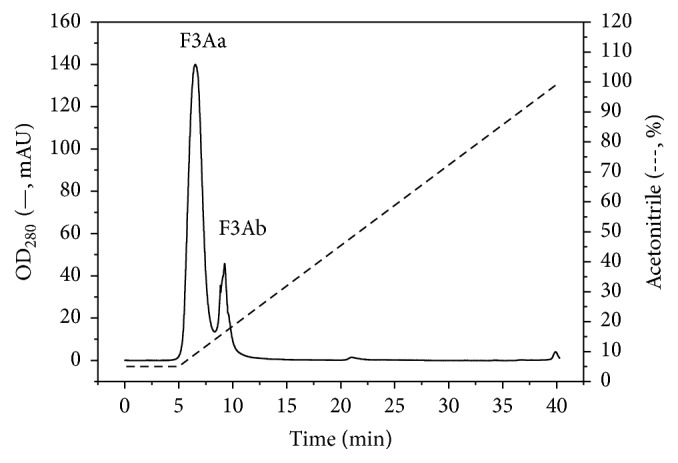
Purification of the bacteriocin produced by* L. pentosus* JL-1 by semipreparative HPLC.

The bacteriocin, pentocin JL-1, is one of the secondary metabolites of* L. pentosus* JL-1 after a 72 h incubation. In order to purify this bacteriocin, macroporous resin, cation-exchange, gel filtration, and semipreparative HPLC were used. During the purification process, its antibacterial activity against MRSA GIM 1.771 was evaluated. The crude bacteriocin was collected using macroporous resin D4020 and then was purified by SP-Sepharose Fast Flow. Three fractions F1, F2, and F3 were collected, and the fraction F3 had antibacterial activity against MRSA GIM 1.771 (see Figure S1 in Supplementary Material available online at https://doi.org/10.1155/2017/7657190), with a specific activity reaching up to 432 IU/mg (Table 3). The active fraction F3 was subsequently purified with Ultrahydrogel TM 250 gel filtration chromatography (Figure S2). In this step, three fractions F3A, F3B, and F3C were collected and their antibacterial activities were tested. The highest antibacterial activity fraction of F3A was collected for further purification by semipreparative HPLC, as shown in Figure 2. The fraction F3Aa had significantly higher antibacterial activity against MRSA GIM 1.771 than F3Ab (*p* < 0.05). F3Aa was repurified by analytical HPLC (data not shown), and the active fraction was collected and lyophilized for MS detection. The purification process and antibacterial activity are listed in Table 3. After the four-step purification, pentocin JL-1 was purified 70.7-fold at a yield of 4.7%. In previous studies, pentocin SJ-65 produced by* L. pentosus* SJ65 was purified 52-fold at a yield of 8% [33], bacteriocin KU24 produced by* L. lactis* KU24 was purified 24.58-fold [25], and plantaricin ZJ5 produced by* L. plantarum* ZJ5 was purified 139.5-fold at a yield of 1.7% [13].

The yield of pentocin JL-1 can be enhanced by optimizing the production conditions. In addition to incubation temperatures, initial pH values, inoculum density, loading volume, and culture medium optimization, coculture with other strains and autoinduction by a signal peptide produced by the strain itself are effective ways to improve the yield of bacteriocins. For example, at low cell densities, gassericin E was produced by* L. gasseri* EV146 after the addition of the supernatant from a previous bacteriocin-producing EV1461 culture (autoinduction) or through cocultivation with several other Gram-positive strains (inducing bacteria) [34]. In addition, genetic engineering is a strategy to enhance the production of bacteriocins. In recent years,* L. plantarum*,* L. lactis*,* L. sakei, S. thermophilus, *and various other LAB have been used as hosts for heterologous expression [32, 35, 36].

In addition, from the purification process, we observed that pentocin JL-1 is a cationic and hydrophobic peptide, which was shown by cation-exchange, hydrophobic-interactions, and C_18_ reverse-phase HPLC (C_18_ RP-HPLC). Many bacteriocins such as bacteriocin KU24 [25], bacteriocin VJ13 [37], plantaricin ZJ5 [13], and other LAB bacteriocins share similar properties. Thus, the purification strategies can be compared and referenced.

### 3.3. Mass Spectrometry and Amino Acid Sequence

**Figure 3:**
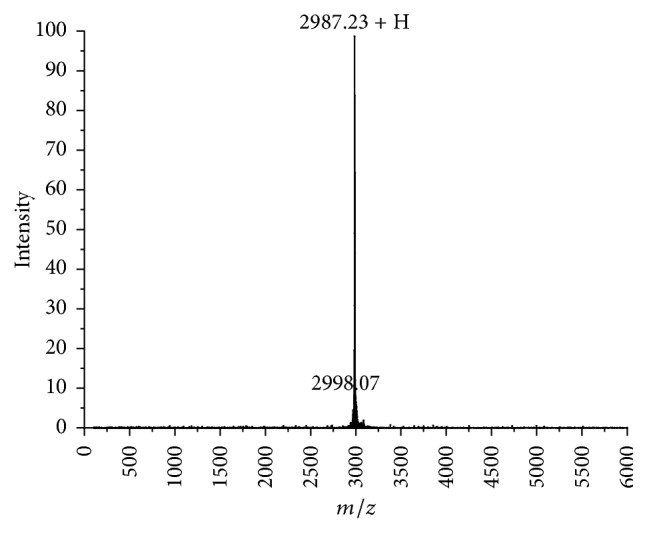
MALDI-TOF-MS of analytical HPLC purified pentocin JL-1.

Pentocin JL-1 was identified by MALDI-TOF-MS. The results indicated that the purified pentocin JL-1 has a molecular mass of 2987.23 Da (Figure 3), which was different from those of the bacteriocins produced by* L. pentosus* reported previously, including the bacteriocin from* L. pentosus* RL2e of around 20 kDa [29], the bacteriocin B231 produced by* L. pentosus* of about 5 kDa [30], pentocin C50-6of about 2.5 kDa [31], and pentocin 31-1 of 5,592.225 Da [38]. In addition, a variety of new bacteriocins produced by LAB has been successfully purified and characterized in the past few years. The molecular masses of the majority plantaricins is >3.0 kDa, although some smaller plantaricins have also been reported, such as plantaricin DL3 (2.1 kDa) [39] and ZJ008 (1334.77 Da) [11]. However, to the best of our knowledge, the present study is the first report of a pentocin with a molecular mass of 2987.23 Da. In addition, six of the twenty-five amino acids of pentocin JL-1 could be identified by Edman degradation and the sequence is VAKVAR. Further sequencing failed probably due to the presence of a modified residue or some rare amino acids in the peptide that prevented cleavage by the Edman's reagent. The sequence showed no homology with other known bacteriocins using protein BLAST against the GenBank database (https://blast.ncbi.nlm.nih.gov/Blast.cgi). Thus, pentocin JL-1 may be a novel LAB bacteriocin. To obtain more detailed information, further chemical and mass spectrometry techniques needed to be performed. In addition, the complete genome sequencing of our* L. pentosus* will be performed to gain insights into the genetic elements involved in bacteriocin production in the future.

### 3.4. Stability against pH, Temperatures, and Enzymes

The effects of pH, temperatures, and enzymes on the antibacterial activity of the bacteriocin were determined (Table 4). No significant differences in antimicrobial activities were found from pH 5 to pH 7 (*p* > 0.05). However, the antimicrobial activity decreased significantly when the pH decreased from 4 to 2 and increased from 8 to 10 (*p* < 0.05). Some other bacteriocins have also been reported to have a similar pH stability [21, 40]. It may be that, at extreme pH values, strong intramolecular electrostatic interactions cause a partial or total loss of activity [41]. However, even at pH 2 and pH 10, pentocin JL-1 still retained 65.69% and 52.30% of antibacterial activity, respectively, which indicates that this bacteriocin may be used in most food.

At different temperatures, pentocin JL-1 was stable, and no significant differences were detected from −20°C to 100°C (*p* > 0.05). An antibacterial activity of 84.10% remained even after autoclavation (121°C, 15 min). This thermostable characteristic makes pentocin JL-1 suitable for use in a sterilization process.

When purified pentocin JL-1 was treated with different hydrolytic enzymes, its inhibitory action was significantly reduced by treatment with proteinase K and trypsin (*p* < 0.05). However, the inhibitory action was completely abolished by treatment with pepsin and alkaline protease. Thus, pentocin JL-1 has a proteinaceous nature like most other bacteriocins [11, 13, 19, 21].

### 3.5. Inhibitory Spectrum

The inhibitory spectrum of pentocin JL-1 is shown in Tables 1 and 2. As shown in Table 1, pentocin JL-1 was inhibitory against both Gram-positive and Gram-negative bacteria. Among the indicator species, the bacteriocin showed the highest activity against the Gram-positive bacteria* L. casei *ATCC 393 and MRSA GIM 1.771 and the Gram-negative bacteria* Shigella dysenteriae *CGMCC 1.1869 and* E. coli* O157:H7 GIM 1.707, with a diameter of inhibition zones 20–25 mm. In addition, it inhibited* Bacillus subtilis* CGMCC 1.1627,* Enterococcus faecalis* ATCC 51575,* Listeria monocytogenes *ATCC 19112,* Micrococcus luteus* CGMCC 1.2299, and* Pseudomonas aeruginosa* CGMCC 1.1785. However, pentocin JL-1 had no inhibitory activity against* L. acidophilus *ATCC 314 and* Vibrio parahaemolyticus *GIM 1.306. Many pentocins produced from* L. pentosus *have been studied against a variety of Gram-positive and Gram-negative bacteria and the fungi* Candida albicans *[18, 29–31]. However, none have been reported to have an anti-MRSA activity, except for pentocin JL-1 in our study.

In particular, besides MRSA GIM 1.771, pentocin JL-1 could also inhibit 6 strains of multidrug-resistant* S. aureus* isolated from pork in our lab (Table 2). Not only can multidrug-resistant* S. aureus *produce toxins, but also the transfer of resistance to other pathogenic bacteria of humans is a potential threat [42]. In addition, the MIC of pentocin JL-1 against the indicator strain MRSA GIM 1.771 was 7.5 *μ*g/mL. It has been reported that plantaricin Pln-1 inhibits MRSA with a MIC of 180 ± 20 *μ*g/mL [43]. The MIC of lactocin XN8-A against* S. aureus *ATCC 29213 is 6.85 *μ*g/mL, but this is not an MRSA strain [21]. Lactocin XN8-A has also antibacterial activity against pork-derived multidrug-resistant* S. aureus*, but this study did not show the MIC data [21]. In our study, pentocin JL-1 exhibited a broad inhibitory spectrum, a low MIC against MRSA, and a high antimicrobial activity against multidrug-resistant* S. aureus*, representing a potential biopreservative in the food industry. Thus, further study was needed to identify its mode of action.

### 3.6. Mode of Action of Pentocin JL-1

#### 3.6.1. Growth Curve and Time-Killing Kinetics

**Figure 4:**
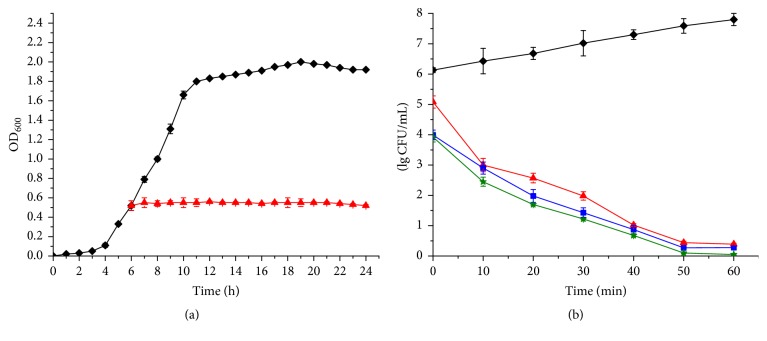
Effects of pentocin JL-1 on intact cells. (a) The effects of pentocin JL-1 on MRSA GIM 1.771 growth and (b) time-killing kinetics by pentocin JL-1. Control (solid diamond); 1x MIC (solid triangle); 2x MIC (solid square); 3x MIC (solid star).

Figure 4(a) shows that the growth curve of MRSA GIM 1.771 for 24 h is typical. However, once 1x MIC of pentocin JL-1 was added, after 6 h (the exponential phase), the OD_600_ values were nearly stable. This means that pentocin JL-1 inhibited the growth of MRSA GIM 1.771 with no clear evidence of cell lysis. However, the time-killing curve showed that when 1x MIC, 2x MIC, and 3x MIC of pentocin JL-1 were added, respectively, a significant downward tendency in the viable count was observed and was dose-dependent to some extent (Figure 4(b)). Additionally, an instantaneous killing action occurred at 0 h by addition of 1x MIC, 2x MIC, and 3x MIC of pentocin JL-1 with 1.05, 2.14, and 2.21 log_10_ reduction, respectively. These results indicate that pentocin JL-1 had a bactericidal activity against MRSA GIM 1.771. Lactocin XN8-A [21], enterocin SN11 [44], and plantaricin ZJ008 [11] have also been reported to have similar bactericidal properties.

#### 3.6.2. PMF

**Figure 5:**
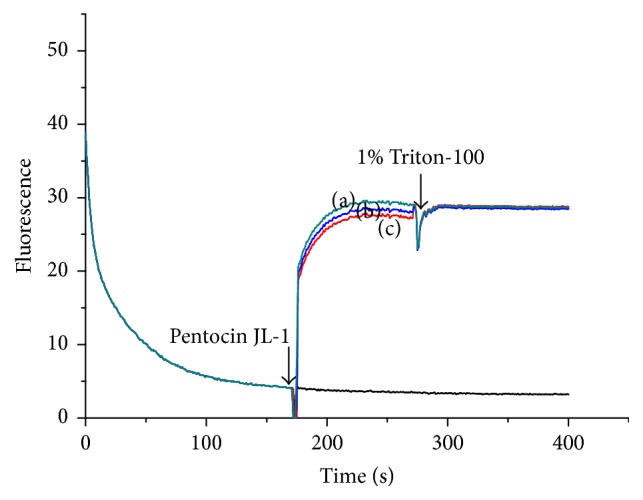
Analysis of ΔΨ of MRSA GIM1.771 cells. MRSA GIM 1.771 cells were treated with 3x MIC (a), 2x MIC (b), and 1x MIC (c) pentocin JL-1, respectively. 1% Triton X-100 was added as ΔΨ 100% dissipation and 0.05% (*w/v*) acetic acid was used as the negative control.

**Figure 6:**
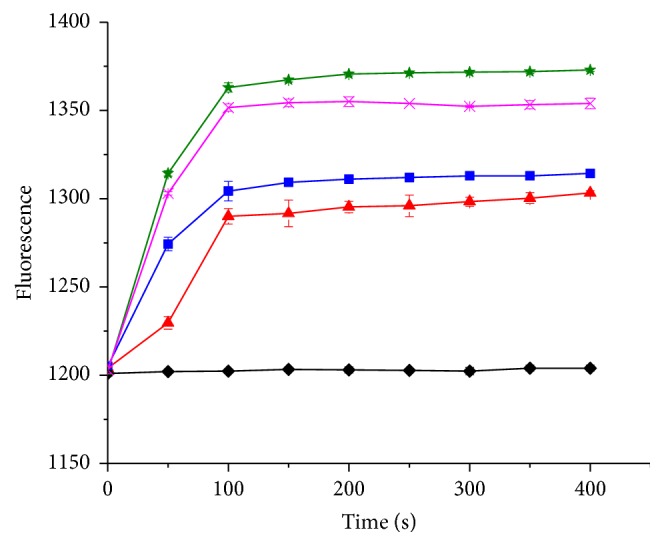
Analysis of ΔpH of MRSA GIM 1.771 cells. MRSA GIM 1.771 cells were treated with 3x MIC (solid star), 2x MIC (solid square), and 1x MIC (solid triangle) pentocin JL-1, respectively. 1% Triton X-100 (cross) was added as ΔpH 100% dissipation and 0.05% (*w/v*) acetic acid (solid diamond) was used as the negative control.

As many bacteriocins are assumed to kill the target microorganism via permeabilization of the cell membrane [45], the effect of pentocin JL-1 on the membrane integrity of MRSA GIM 1.771 intact cells was determined by the membrane potential sensitive dye DisC_2_(5) (Figure 5) and the transmembrane pH gradient fluorescent probe BCECF (Figure 6). As shown in Figure 5, when the reduction of fluorescence was stable, the accumulated dye in the membrane interior of energized cells was quenched. After a stable signal was observed, addition of the pentocin JL-1 (indicated by the first arrow in Figure 5) caused a rapid increase in fluorescence due to the collapse of the ion gradients that generate the membrane potential [46]. After the fluorescence stabilization, 1% Triton X-100 (indicated by the second arrow in Figure 5) was subsequently added, and the results indicated the 100% dissipation of the membrane potential. As shown in Figure 5, addition of 1% Triton X-100 only caused a small further increase in fluorescence for curves (b) and (c) and was nearly flat for curve (a), showing that pentocin JL-1 causes cell membrane permeabilization. Additionally, the ability of pentocin JL-1 to disturb the membrane barrier was dose-dependent. However, the fluorescence of the control sample, to which 0.05% (*w/v*) acetic acid was added, did not indicate any increase or decrease during the 400 s experiment.

Once BCECF AM is absorbed into the cell membrane, it is cleaved by an esterase into BCECF, which is a fluorescent probe that indicates a transmembrane pH gradient. As shown in Figure 6, when the samples were exposed to 1x MIC, 2x MIC, and 3x MIC of pentocin JL-1, the fluorescence of BCECF increased within 100 s and increased slightly at the later time points, indicating that ΔpH of MRSA GIM 1.771 was rapidly dissipated by pentocin JL-1. ΔpH was stable in the control sample after a 400 s incubation. However, the final fluorescence of the 1x MIC and 2x MIC treated samples was lower than that of the samples exposed to 1% Triton X-100. These results suggest that ΔpH is incompletely dissipated by 1x MIC and 2x MIC of pentocin JL-1.

In general, cationic bacteriocins initially interact with the anionic cell membrane through electrostatic attraction [47]. Then, bacteriocins permeabilize the cell membrane to dissipate ΔΨ and ΔpH, which constitute the PMF of the cells [48]. Finally, bacteriocins have bacteriostatic or bactericidal effects. In our study, pentocin JL-1 dissipated ΔΨ and ΔpH of MRSA GIM 1.771. This result shows that the addition of pentocin JL-1 leads to the dissipation of the PMF of MRSA GIM 1.771 due to the loss of vital ion gradients and suggests that the membrane is the target of pentocin JL-1. In addition, the dissipation of the PMF was dose-dependent. Similar dose-dependent cell membrane potential dissipation results have also been shown for other bacteriocins such as aureocin A53 [49] and Pln EF [26].

ΔΨ and ΔpH dissipations were nearly complete within 100 s, which shows that the membrane permeabilization caused by pentocin JL-1 is a relatively rapid process. This is in accordance with the well-known antibiotic peptide clavanin [46], which is a membrane-targeted and dose-dependent peptide. However some other membrane-targeted bacteriocins have a gradual process of membrane potential dissipation [26, 50].

#### 3.6.3. SEM

**Figure 7:**
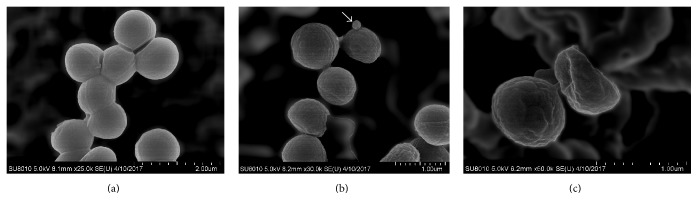
Scanning electron micrographs of MRSA GIM 1.771 cells. (a) Untreated control cells; (b) and (c) 1x MIC of pentocin JL-1 treated cells.

SEM was used to further demonstrate the membrane damage of MRSA GIM 1.771 caused by pentocin JL-1. Morphological changes of MRSA GIM 1.771 after 10 min exposure to 1x MIC of pentocin JL-1 are presented in Figure 7. Compared with the smooth surface of the control cells with integrated and plump cell structures (Figure 7(a)), cell membrane disruption and deformation with shrinking and cavities were observed on the cell surface of cells treated with pentocin JL-1 (Figures 7(b) and 7(c)). In addition, blebs (the arrow indicated in Figure 7(b)) protruded into the cell surface, which also shows that pentocin JL-1 acts on the cell surface. Blebs are a kind of vesicles, which are induced by external stimulus and might play an important role in cell-to-cell communication [26]. Cell membrane damage was clear in MRSA GIM 1.771 treated with pentocin JL-1 and is a typical characteristic caused by bacteriocins [26]. Similar membrane damage has also been reported for nisin, pediocin, and Pln EF-treated cells [26, 51, 52].

## 4. Conclusions

In the present study, the bacteriocin pentocin JL-1, produced by* L. pentosus *isolated from the intestinal tract of* C. punctatum*, was purified and found to have a molecular mass of 2987.23 Da. It is sensitive to proteinase K, trypsin, pepsin, and alkaline protease, indicating that it has a proteinaceous nature. Also, this bacteriocin has a broad inhibitory spectrum, is thermostable, and stable over a pH range of 5–7. Hence, pentocin JL-1 appears to have promising potential as a biopreservative in the food industry, especially for controlling multidrug-resistant* S. aureus*. Additionally, our results show that the cell membrane is the target of pentocin JL-1 against MRSA GIM 1.771, causing a loss of PMF in only a few minutes, and that it has a drastic impact on the structure and integrity of the MRSA GIM 1.771 cell that finally leads to cell death, which was indicated by the growth curve and time-killing kinetics. In further studies, more detailed information on the mode of action, the exact amino acid sequence, and the structure of pentocin JL-1 will be addressed.

## Supplementary Material

Figure S1: Purification of the bacteriocin produced by *L. pentosus* JL-1 by SP-Sepharose Fast Flow chromatography.
Figure S2: Purification of the bacteriocin produced by *L. pentosus* JL-1 by Ultrahydrogel TM 250 gel filtration chromatography.

## Figures and Tables

**Table 1:** Inhibitory spectrum of pentocin JL-1.

Indicator strains	Source	G^+^/G^−^	Antimicrobial activity
*Lactobacillus acidophilus*	ATCC314	G^+^	−
*Lactobacillus casei*	ATCC393	G^+^	+++
*Bacillus subtilis*	CGMCC1.1627	G^+^	++
MRSA	GIM1.771	G^+^	+++
*Enterococcus faecalis*	ATCC51575	G^+^	++
*Listeria monocytogenes*	ATCC19112	G^+^	++
*Micrococcus luteus*	CGMCC1.2299	G^+^	++
*Vibrio parahaemolyticus*	GIM1.306	G^−^	−
*Pseudomonas aeruginosa*	CGMCC1.1785	G^−^	+
*Shigella dysenteriae*	CGMCC1.1869	G^−^	+++
*Escherichia coli* O157:H7	GIM1.707	G^−^	**+++**

Inhibition zone in diameter (mm): +++: 20–25; ++: 15–19; +: 10–14; −: no inhibitory activity (including the 8 mm diameter of each well). ATCC, American Type Culture Collection, Virginia, USA; CGMCC, China General Microbiological Culture Collection Center, Beijing, China. GIM, Guangdong Microbiology Culture Center, Guangdong, China.

**Table 2:** Pentocin JL-1 activity against multidrug-resistant *S. aureus*.

Indicator strains	Isolation sources	Inhibitor zone (mm)	Resistance antibiotics^a^
Multidrug-resistant* S. aureus* 1	Pork	23.8 ± 0.8	FOX, TET
Multidrug-resistant* S. aureus* 2	Pork	22.5 ± 1.2	FOX, GEN
Multidrug-resistant* S. aureus* 3	Pork	24.3 ± 0.7	FOX, TET, GEN
Multidrug-resistant* S. aureus *4	Pork	22.4 ± 0.6	FOX, TET, C
Multidrug-resistant* S. aureus *5	Pork	23.9 ± 1.5	FOX, TET, GEN, C
Multidrug-resistant* S. aureus *6	Pork	23.7 ± 0.6	CIP, FOX, C, SXT, TET, GEN

^a^FOX, cefoxitin; TET, tetracycline; GEN, gentamicin; C, chloramphenicol; CIP, ciprofloxacin; SXT, trimethoprim-sulfamethoxazole.

**Table 3:** Purification and activity of the bacteriocin produced by *L. pentosus* JL-1.

Samples	Total protein (mg)	Total bacteriocin activity (IU)	Specific activity (IU/mg)	Purification (fold)	Yield (%)
Supernatant	2690	100440	37	1.0	100.0
Macroporous resin D4020	460	49740	108	2.9	49.5
Cation exchange	47	20320	432	11.7	20.2
Gel chromatography	12	11090	924	25.0	11.0
C_18_ RP- HPLC	1.8	4710	2617	70.7	4.7

**Table 4:** Stability of pentocin JL-1 against pH, temperatures and enzymes.

Treatment	Residual inhibitory activity (inhibition zone diameter, mm)	Residual inhibitory activity (%)
*pH value*		
Control (5.5)	23.9 ± 0.7	100.00
2.0	15.7 ± 1.4	65.69^*∗*^
3.0	16.2 ± 1.8	67.78^*∗*^
4.0	16.8 ± 1.2	70.29^*∗*^
5.0	23.9 ± 0.4	100.00
6.0	23.9 ± 0.8	100.00
7.0	23.6 ± 0.6	98.74
8.0	14.6 ± 0.3	61.09^*∗*^
9.0	12.9 ± 0.9	53.97^*∗*^
10.0	12.5 ± 1.6	52.30^*∗*^
*Temperature*		
Control	23.9 ± 0.7	100.00
−20°C, 1 h	22.2 ± 1.0	93.00
4°C, 1 h	23.8 ± 1.2	99.58
30°C, 1 h	23.9 ± 0.2	100.00
60°C, 1 h	23.6 ± 0.8	98.74
100°C, 1 h	22.6 ± 0.8	94.52
121°C, 15 min	20.1 ± 0.9	84.10^*∗*^
*Enzyme*		
Control	23.9 ± 0.7	100.00
Proteinase K (pH 7.5, 55°C)	17.3 ± 1.2	72.38^*∗*^
Trypsin (pH 8.0, 37°C)	15.7 ± 1.2	65.69^*∗*^
Pepsin (pH 1.8, 37°C)	0.0 ± 0.0	0.00^*∗*^
Alkaline protease (pH 8.6, 50°C)	0.0 ± 0.0	0.00^*∗*^

^**∗**^The decrease being considered statistically significantly (*p* < 0.05).
