# Supplementary material for: Purification, Characterization, and Mode of Action of Pentocin JL-1, a Novel Bacteriocin Isolated from Lactobacillus pentosus, against Drug-Resistant Staphylococcus aureus
Source: Biomed Res Int. 2017 Nov 29;2017:7657190. doi: 10.1155/2017/7657190 (PMC5733122; doi:10.1155/2017/7657190)
Supplement: Supplementary file 1 — Figure S1: Purification of the bacteriocin produced by L. pentosus JL-1 by SP-Sepharose Fast Flow chromatography. Figure S2: Purification of the bacteriocin produced by L. pentosus JL-1 by Ultrahydrogel TM 250 gel filtration chromatography. [file 7657190.f1.pdf]

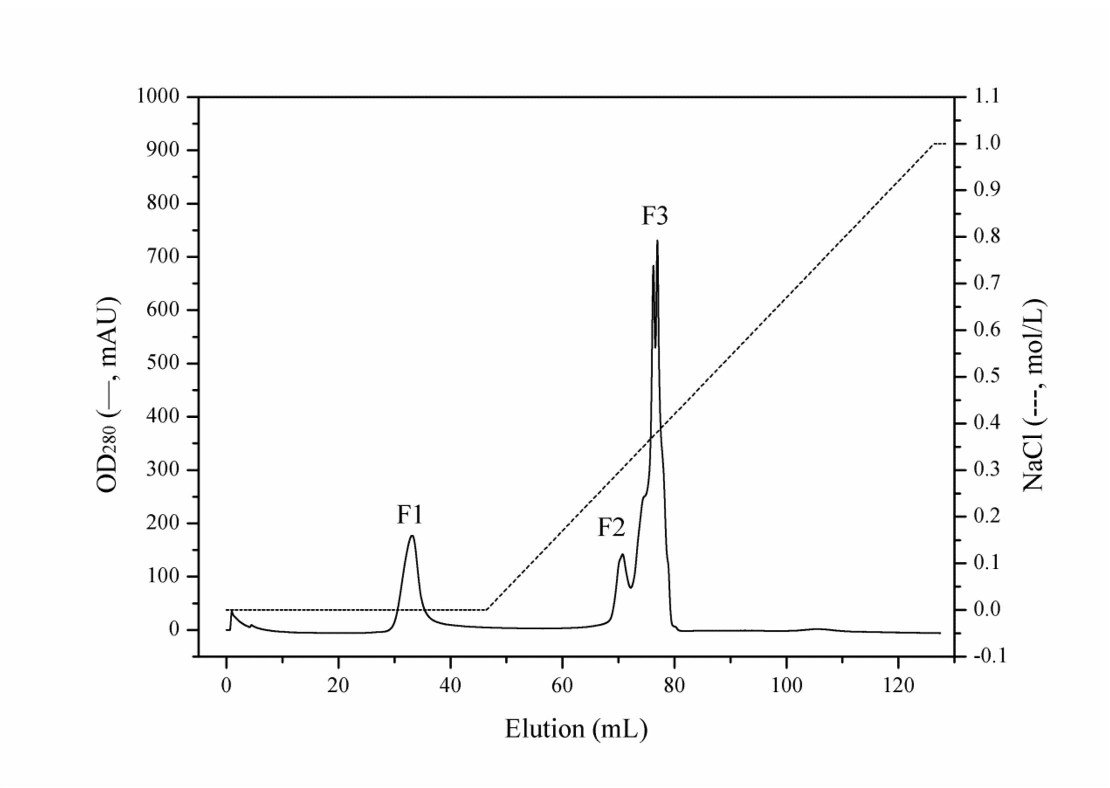

FIGURE S1: Purification of the bacteriocin produced by *L. pentosus* JL-1by SP-Sepharose Fast Flow chromatography.

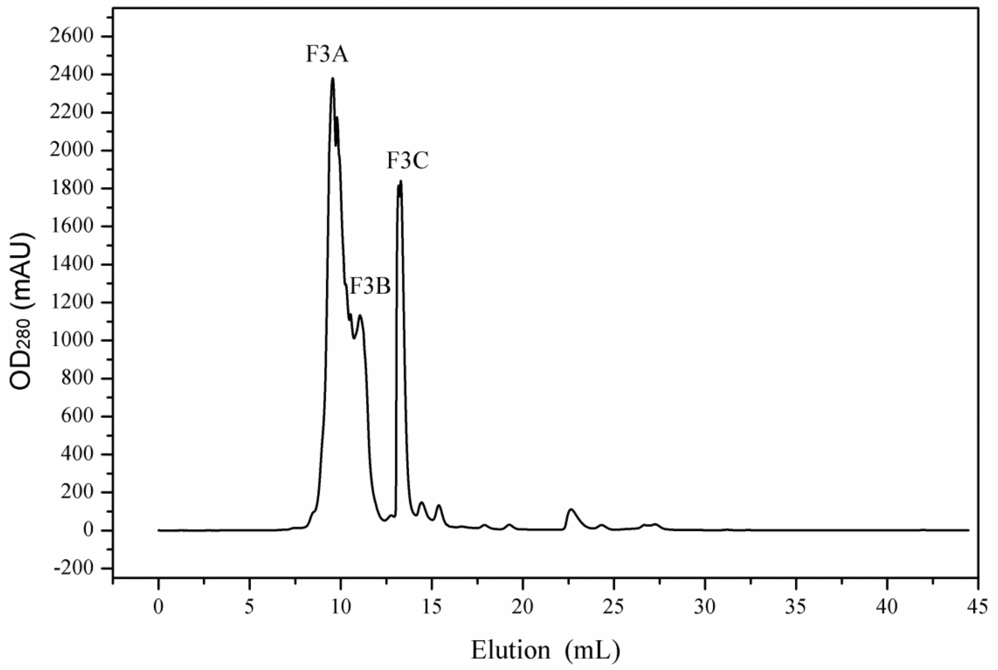

FIGURE S2: Purification of the bacteriocin produced by *L. pentosus* JL-1by Ultrahydrogel TM 250 gel filtration chromatography.
